# Use of organ-on-chip devices and single particle ICP-MS for assessing dynamic in vitro bioavailability of silver and titanium dioxide nanoparticles from foodstuff

**DOI:** 10.1007/s00604-026-08062-y

**Published:** 2026-04-24

**Authors:** Osvaldo Beltrán-Osuna, Juan José López-Mayán, Lucía Gómez-Cibeira, Oier Jurado-Martín, Alejandro Ogando-Cortés, Raquel Domínguez-González, Pablo Taboada-Antelo, Pilar Bermejo-Barrera, Antonio Moreda-Piñeiro

**Affiliations:** 1 Colloids and Polymer Physics Group. Institute of Materials (iMATUS), Department of Particle Physics, Faculty of Physics, University of Santiago de Compostela, Santiago de Compostela, Spain; 2 Trace Elements, Spectroscopy, and Speciation Group (GETEE), Institute of Materials (iMATUS), Department of Analytical Chemistry, Nutrition and Bromatology, Faculty of Chemistry, University of Santiago de Compostela, Santiago de Compostela, Spain

**Keywords:** Organ on a chip, Cellular transport, Inorganic nanoparticles, Foodstuff

## Abstract

The most common way to study the cellular transport of nutrients and pollutants when simulating intestinal absorption is to use static in vitro models. However, these approaches are limited by the lack of dynamism in simulating the intestinal absorption process, and they fail to consider the stress created by fluid during this process. The development of micro-physiological systems, such as organ-on-a-chip (OoC), enables the dynamics of the process to be incorporated into the simulation, offering a more realistic approach. This research has studied the potential for using an OoC with Caco-2 cells to simulate the intestinal absorption of inorganic silver and titanium dioxide nanoparticles in food additives (E171) and food, e.g. sugar pearls containing the food additive E174 and seafood, -seaweed and mussels which were exposed to nanoparticles in controlled laboratory trials. A viable cell monolayer was achieved under flow conditions for 96 h using the commercial μ-Slide I Luer 3D microfluidic device. This made possible to carry out cell transport experiments on the bio-accessible fractions obtained from the samples under study. The nanoparticle content (particle number concentration) and particle size distribution were obtained using inductively coupled plasma mass spectrometry working in the single particle mode (spICP-MS). This technique is of great importance to monitor particle size changes given the agglomeration and/or dissolution phenomena that nanoparticles undergo depending on the surrounding environment. To study these processes, complementary techniques such as transmission electron microscopy, were used.

**Graphical Abstract:**

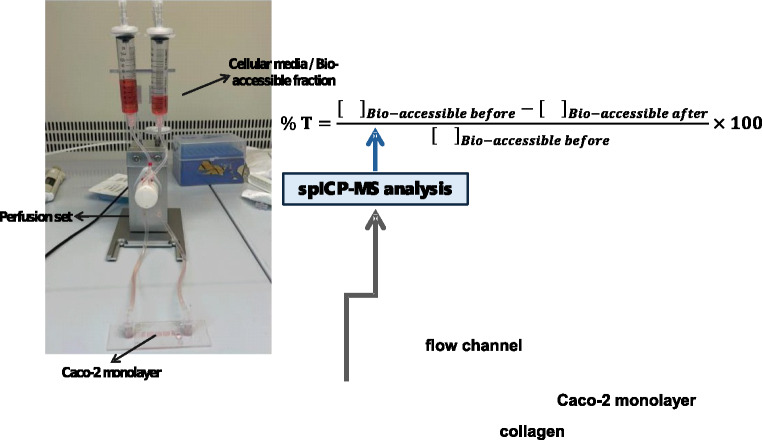

**Supplementary Information:**

The online version contains supplementary material available at 10.1007/s00604-026-08062-y.

## Introduction

Inorganic nanoparticles (NPs) and nanomaterials (NMs) are widely used, and applications derived from these new materials have revolutionised sectors such as agriculture, electronics, energy and food [1]. Silver nanoparticles (Ag NPs) and titanium dioxide nanoparticles (TiO_2_ NPs) are among the most widely used NPs in the food industry [2]. The demonstrated antimicrobial and antifungal properties of Ag NPs make them useful for packaging, as they prevent microbial attack and increase the shelf life of food [3]. The benefits of TiO_2_ NPs are attributed to their antimicrobial activity (they act as a photocatalytic antibacterial agent), their anti-caking effect in food packaging, and their whitening properties. Macro TiO_2_ and TiO_2_ NPs are included in the food additive E171 as a whitening pigment [3, 4]. Therefore, there is particular concern about NPs as emerging pollutants and potential hazards to human health due to their various applications in the food industry [5, 6]. In this scenario, the EU banned the use of the food additive E171 in 2022 due to evidence of the genotoxicity of TiO₂ NPs [7]. However, E171 can still be used as an ingredient in medicines and supplements.

The main route of human exposure to food-derived NPs is through oral ingestion. Once in the gastrointestinal tract, NPs encounter a variety of environments and interact with different biological fluids, which can result in their agglomeration, aggregation, or dissolution [8]. Until now, the primary models used to study the release and transformation of nutrients from food in the gastrointestinal tract have been in vivo models (animals [9–11] or humans). In vivo tests on animals have several limitations. For example, they may not accurately represent human physiology, as well as they are also expensive and raise ethical concerns [12]. An alternative approach is to use in vitro models in which the oral, gastric and intestinal phases can be simulated by controlling parameters such as temperature, pH, incubation time and the composition of gastrointestinal fluids. The major advantage of both static and dynamic in vitro models is their high repeatability, which allows the bio-accessibility of substances to be compared under different conditions**.** For example, the bio-accessibility of such a compound can be compared when ingested alone or when contained in a food, as well as when contained in different foods.

In vitro tests can be divided into cellular and non-cellular tests. Non-cellular models enable the bio-accessibility of a compound to be studied under different conditions and provide information for the risk assessment of chemical species and their transformations during simulated gastrointestinal processes. This is particularly important when dealing with NMs, as changes in their size during the gastrointestinal process may alter their classification as hazardous or not to human health [5, 13–16]. Cellular assays use cell monolayers to simulate absorption processes that can occur in the intestine, enabling the bioavailability of a substance to be estimated. Most cellular models are based on static approaches to assess the permeability, transport and absorption of nutrients and pollutants across Caco-2 cells [17–19] cultures and Caco-2/HT29 [20–22] cocultures. Recently, bioavailability studies of Ag and TiO_2_ NPs in seafood have been developed using this approximation [23, 24], which implies that the bio-accessible fraction containing the nanoparticles is in static contact with the cell monolayer for the estimated duration required to simulate intestinal absorption. However, according to a recent review by Ritarossi et al. [25], the limitations of static in vitro assays can be overcome by using intestinal organoids, reconstructed intestinal tissues under fluidic and mechanically stimulated conditions, all of which better simulate the complexity of intestinal architecture.

Consequently, there is a growing interest in tissue engineering and microfabrication techniques for developing dynamic cellular systems that can more accurately and consistently mimic the intestinal absorption/transport mechanism. These systems, also known as micro-physiological systems or Organ-on-a-Chip [26–28] (OoC) microdevices, allow for a more realistic simulation of the interaction between fluids (i.e. the bio-accessible fraction derived from gastrointestinal digestion of food) and cells, since fluid flow creates constant stress (i.e. a mechanical force) that significantly affects the physiology, structure and function of cells [29]. Although OoC systems are an innovative technology and a novel field that is still being investigated, significant progress has been made in developing commercial OoC systems that simulate intestinal functions (gut-on-a-chip). Current applications have focused on pharmacokinetic studies of new chemotherapeutic agents to establish gastrointestinal solubility and permeability [30], and on drugs such as ifosfamide and verapamil [31], antipyrine, ketoprofen and digoxin [32] and peptide formulations that enhance the intestinal permeability of certain drugs [33]. To date, however, there have been no published applications of these dynamic systems for assessing the bioavailability of essential compounds and contaminants, nor for nanomaterials. The current study therefore aimed to apply/adapt a commercial OoC system (µ-Slide I Luer 3D chip by Ibidi) to assess the bioavailability of Ag NPs and TiO_2_ NPs in standards and foodstuffs (seafood and confectionery products).

## Materials and methods

### Nanoparticles determination by spICP-MS

The determination of NPs (i.e. particle number concentration and size distribution) was performed using spICP-MS (NexION 2000 with Syngistix™ Nano Application software, PerkinElmer, Massachusetts, USA). Determination of Ag NPs was carried out under standard conditions, whereas TiO_2_ NP determinations were performed using the dynamic reaction cell (DRC) mode (NH₃ as the collision gas and an ammonia cluster ^48^Ti(NH)(NH_3_)_4_ at m/z 131) [34]. A detailed description of the reagents and materials used in the spICP-MS analyses, as well as operating conditions, can be found in the Electronic Supplementary Material (ESM) section.

### Mussel, seaweed, and confectionery products

The samples used in the current study consisted of mussels (*Mytilus edulis*) that had previously been exposed to Ag NPs and TiO₂ NPs under controlled conditions: 15 nm PVP-stabilized Ag NPs at a concentration of 1.0 mg L^−1^ for 28 days and 25 nm citrate-stabilized TiO₂ NPs 1.0 mg L^−1^ for 28 days). The red seaweed dulse (*Palmaria palmata*) and the green seaweed sea lettuce (*Ulva sp.*) were also exposed to Ag and TiO₂ NPs under controlled conditions (15 nm PVP- stabilized Ag NPs at 1.0 mg L^−1^ for 28 days, and 25 nm citrate-stabilized TiO₂ NPs at 1.0 mg L^−1^ for 28 days) [35, 36].

The confectionery products consisted of small and medium-sized commercial sugar pearls and sheets containing the food additive E174, which came from different manufacturers.

### Sample pretreatment procedure for NPs extraction from foodstuff

Due to the variety of foodstuffs analysed (molluscs, seaweed, and confectionery products) and the different types of nanoparticles studied (TiO_2_ and Ag NPs), various extraction procedures were applied, including enzymatic hydrolysis and the use of tetramethylammonium hydroxide (TMAH) [37–39]. A detailed description of each procedure, along with the reagents and materials used, can be found in the ESM section.

### *Invitro *gastrointestinaldigestion

**Fig. 1:**
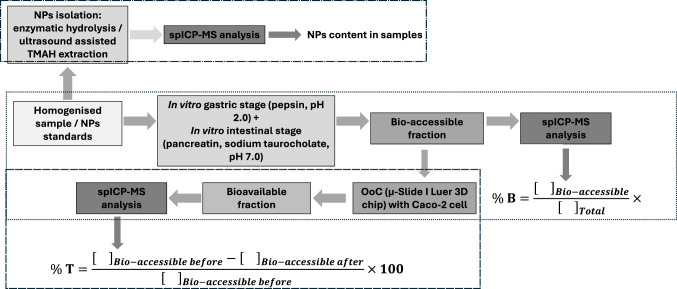
Workflow of the overall procedure (assessment of Ag NPs and TiO_2_ NPs in samples, and Ag NPs and TiO_2_ NPs bio-accessibility, and cellular transport)

The in vitro gastrointestinal digestion process consists of two successive stages, in which gastric and intestinal conditions are simulated. Figure 1 illustrates the workflow for each sample (confectionery products, seaweed, and mussels), as well as for the standards. A detailed description of the procedure, as well as details of the equipment and reagents used, can be found in the ESM section.

The bio-accessibility ratio (% B), defined as the proportion of NPs that are not degraded during gastrointestinal digestion and become available for intestinal absorption, was calculated using Eq. 1

1 $${\% Bio}-\mathrm{accessibility}=\frac{{\left[\hspace{0.17em}\right]}_{Bio-accessible}}{{\left[\hspace{0.17em}\right]}_{Total}}\times 100$$

where []_Total_ is the TiO_2_ NPs/Ag NPs number concentration after enzymatic hydrolysis and spICP-MS measurement, and []_Bio-accessible_ is the TiO_2_ NPs/Ag NPs number concentration after in vitro bio-accessibility and spICP-MS analysis.

### *Invitro *bioavailability (cellular transport) using an OoC system

Before conducting cellular transport assays using a OoC system, it is necessary to condition the bio-accessible extract before it comes into contact with the cell monolayer. This procedure involves adjusting the osmolarity to 280–300 mOsm kg⁻1, and a description can be found in the ESM section. In addition, the ESM section also details the cell (Caco-2) culture procedure, the description of the μ-Slide I Luer 3D microfluidic chip (Ibidi, Gräfelfing, Germany), the procedure for developing the cell monolayer on the chip under dynamic conditions, the assays for verifying the integrity of the cell monolayer, as well as the optimization of the OoC operating conditions.

Once the cell monolayer had formed, a clamp is placed on the tubing connecting the chip’s inlet and outlet to prevent emptying and air ingress, and the cell culture is then removed from the syringes of the perfusion system. The conditioned bio-accessible fraction is then introduced into the perfusion system and the clamp blocking the inlet and outlet of the chip is removed, allowing the bio-accessible fraction to come into contact with the cell monolayer. The microfluidic system was placed in the incubation chamber (37 °C, 5% CO₂ and 90% humidity) and operated for 2 h. After this time, the pump was stopped, and the bio-accessible fraction was collected from the perfusion system for subsequent analysis.

Bioavailability (i.e. transcellular transport across the intestinal epithelium) of TiO₂ NPs and Ag NPs was obtained by applying Eq. 2.

2 $${\% Transport}=\frac{{\left[\hspace{0.17em}\right]}_{Bio-accessible before}-{\left[\hspace{0.17em}\right]}_{Bio-accessible after}}{{\left[\hspace{0.17em}\right]}_{Bio-accessible before} }\times 100$$

where []_Bio-accessible before_ is the TiO_2_ NPs/Ag NPs number concentration in the bio-accessible fraction before dynamic transport, and []_Bio-accessible after_ is the TiO_2_ NPs/Ag NPs number concentration in the bio-accessible fraction after dynamic transport.

### TEM and HRTEM-EDX analysis of bio-accessible/transport fractions

The bio-accessible fractions, both before and after perfusion, were analysed by HRTEM-EDX. Similarly, the Caco-2 cell monolayer was analysed by TEM. In the first case, the fractions were subjected to a dialysis process followed by ultracentrifugation filtration to ensure the purity of the sample. In the second case, the Caco-2 cells were fixed for subsequent cell sectioning using an ultramicrotome before TEM analysis. The operating conditions, as well as the required reagents and materials for these procedures, are shown in the ESM section.

## Results and discussion

### Bio-accessibility of Ag NPs and TiO_2_NPs

#### ***Bio-accessibility of Ag NPs (40 and 60 nm standards) and TiO***_***2***_*** NPs (50 and 100 nm standards, and food additive E171)***

Standards of two different sizes of Ag and TiO₂ NPs (Table 1), as well as the food additive E171, were subjected to a simulated in vitro gastrointestinal digestion procedure (Sect. "In vitro gastrointestinal digestion"). The bio-accessibility ratio (%B) was then calculated based on the spICP-MS measurements of the bio-accessible fractions obtained.

**Table 1:** Concentrations and sizes of the commercial NPs slurries used to carry out the in vitro gastrointestinal digestion

Ag NPs		TiO_2_ NPs	
Size (nm)	g Ag NPs L^−1^	Size (nm)	g TiO_2_ NPs L^−1^
60	2.0 × 10^–5^	100	6.6 × 10^–4^
40	1.3 × 10^–4^	50	6.5 × 10^–5^
		E171 (nano-/micro-titanium)	5.8 × 10^–4^

As NPs (primarily TiO₂ NPs) tend to agglomerate in the presence of salts and large molecules, the data presented in Figure S1 (ESM), as well as those presented in subsequent figures and tables, refer to Ti and Ag mass rather than TiO₂ and Ag number concentration, respectively. For this calculation, spherical shapes were assumed for both the TiO₂ and the Ag NPs, and the mean diameter of the NPs (obtained from spICP-MS) was used to determine the Ti or Ag mass content in the TiO₂ NPs and the Ag NPs.

As shown in Figure S1A (ESM), the bio-accessibility of Ag NPs does not appear to depend on size (76 ± 10% and 73 ± 4% for 40 nm and 60 nm Ag NPs, respectively), and similar consistent results were also obtained for TiO₂ NPs in Figure S1B (ESM), which were 52 ± 19% and 53 ± 4% for 50 nm and 100 nm TiO₂ NPs, respectively. Statistical analysis was performed (*P* < 0.05; Fischer’s F test for variance comparison and Student’s t test for comparison of %B ratios), and the results confirmed that there were no significant statistical differences in %B when the size of the Ag NPs and TiO₂ NPs varied. The lower %B of the TiO₂ NPs compared to Ag NPs is attributed to particle agglomeration induced by large molecules such as the enzymes used in the simulated gastrointestinal digestion process. This results in a partial loss of TiO₂ NPs during the centrifugation stage. A similar phenomenon must occur with real foodstuffs, whereby some of the released TiO₂ NPs can attach to the food matrix residues.

In the case of Ag NP standards, partial dissolution has been observed under gastrointestinal conditions, as the mean sizes of the bio-accessible fractions from 60 and 40 nm Ag NP standards were 53 ± 4 nm and 35 ± 3 nm, respectively. Higher sizes were obtained for the TiO₂ NPs standards in the bio-accessible fractions, with mean sizes increasing to 148 ± 4 nm for the 50 nm TiO₂ NPs standards (mean size of 78 ± 7 nm in the initial standard suspension) and to 286 ± 11 nm for the 100 nm TiO₂ NPs (mean size of 97 ± 5 nm in the initial standard suspension), respectively. This can be explained by TiO₂ NPs’ tendency to agglomerate in the presence of large molecules and electrolytes, as mentioned previously.

Regarding the food additive E171, low bio-accessibility ratios of 28 ± 4% were found. In this instance, agglomeration is likely to be a more significant factor given that the food additive E171 contains substantial quantities of micro TiO₂ in addition to TiO₂ NPs.

#### Bio-accessibility of Ag NPs from confectionary products containing the food additive E174

Analysis of the Ag NPs in the confectionery products revealed that the Ag NP content, expressed as Ag mass, was 0.236 ± 0.0361 µg g^−1^ and 0.0953 ± 0.0101 µg g^−1^ for samples 1 and 2 (small and medium-sized sugar pearls, respectively). No Ag NPs were detected in the third analysed sample (sample 3), which consisted of fine silvered sheets. The mean size of the isolated Ag NPs (see Sect. "Sample pretreatment procedure for NPs extraction from foodstuff") was found to be 77 ± 2 nm and 60 ± 2 nm for samples 1 and 2, respectively (Table 2).

**Table 2:** Mean sizes of Ag NPs isolated from confectionary products and after the in vitro gastrointestinal digestion

	Mean size (nm)	
	Sample extract^a^	Bio-accessible fraction^b^
Sample 1	77 ± 2	30 ± 3
Sample 2	60 ± 2	34 ± 2

^a^Ag NPs mean size in confectionary products; ^b^Ag NPs mean size after in vitro gastrointestinal digestion

The bio-accessibility of the sugar pearls containing Ag NPs was found to be similar for both samples (69% ± 4% for sample 1 and 73% ± 6% for sample 2), which are close to the values achieved for Ag NP standards (see Figure S1(A), ESM). As can be seen in Table 2, the mean size of the Ag NPs was found to have decreased significantly (by almost half) following in vitro gastrointestinal digestion. These findings demonstrate significant ionisation, that is, degradation of the Ag NPs under gastrointestinal conditions, which is in contrast with the results achieved for the Ag NP standards, with less significant changes in size distribution significant. The lower ionisation of the Ag NP standards (sodium citrate-stabilized Ag NPs) is probably due to their superior stability.

#### ***Bio-accessibility of Ag NPs and TiO***_***2***_*** NPs from seafood***

Regarding cooked seafood (see detail of the culinary process in ESM), Figure S1 (ESM) shows that quite different bio-accessibilities were obtained for Ag NPs from seafood (23 ± 2%, 17 ± 4%, and 98 ± 15% for dulse, mussel, and sea lettuce, respectively), all of them quite different than those obtained for Ag NPs standards and Ag NPs from confectionary products (Sects. "Bio-accessibility of Ag NPs (40 and 60 nm standards) and TiO2 NPs (50 and 100 nm standards, and food additive E171)" and "Bio-accessibility of Ag NPs from confectionary products containing the food additive E174"). Similarly, the bio-accessibility of TiO_2_ NPs varied considerably among the cooked seafood samples (28 ± 4%, 69 ± 9% and 92 ± 8% for dulse, sea lettuce and mussels, respectively) and was also quite different to that observed in TiO_2_ NP standards. These findings suggest that the food matrix exerts a strong influence on the bio-accessible target fractions, a fact that has also been reported in the literature for both trace elements and NPs [40, 41].

The mean sizes of the Ag NPs and TiO_2_ NPs found in the bio-accessible fractions of seafood were similar as those found in the samples after optimised extraction conditions were applied (see Sect. "Sample pretreatment procedure for NPs extraction from foodstuff"), as shown in Table 3. However, the mussel sample was an exception in terms of TiO₂ NPs (Table 3), with lower mean sizes assessed for this type of NPs in the bio-accessible fraction.

**Table 3:** Mean sizes of Ag NPs and TiO_2_ NPs in seafood samples and in in vitro bio-accessible fractions

	Ag NPs mean size (nm)^a^	
	Sample extract^b^	Bio-accessible fraction^c^
Dulse (*Palmaria Palmata*)^d^	22 ± 1	25 ± 1
Sea lettuce (*Ulva sp.*)^d^	31 ± 5	30 ± 1
Mussel (*Mytilus edulis*)^d^	39 ± 1	38 ± 3
	TiO_2_ NPs mean size (nm)^a^	
Dulse (*Palmaria Palmata*)^d^	86 ± 5	77 ± 10
Sea lettuce (*Ulva sp.*)^d^	88 ± 2	81 ± 2
Mussel (*Mytilus edulis*)^d^	96 ± 2	72 ± 2

^a^*n* = 6; ^b^sample after Ag NPs/TiO_2_ NPs isolation; ^c^ bio-accessible fraction (in vitro bio-accessibility process); ^d^ seafood exposed to Ag NPs/TiO_2_ NPs under controlled laboratory conditions

### Bioavailability (cellular transport) of Ag NPs and TiO_2_ NPs by dynamic Organ on aChip

#### ***Cellular transport of Ag NPs (40 and 60 nm standards) and TiO***_***2***_*** NPs (50 and 100 nm standards, and food additive E171)***

**Fig. 2:**
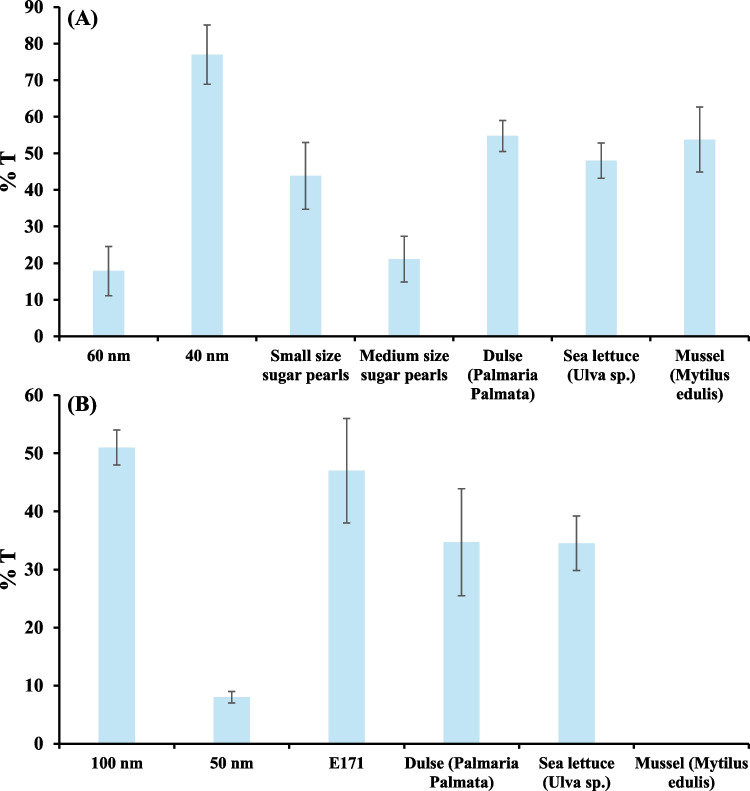
Cellular transport (bioavailability) of Ag NPs in Ag NPs standards (60 and 40 nm), confectionery products (sugar pearls) and exposed seaweeds (dulse and sea lettuce) and mussels (**A**), and cellular transport (bioavailability) of TiO_2_ NPs in TiO_2_ NPs standards (100 and 50 nm), food additive E171, and exposed seaweeds (dulse and sea lettuce) and mussels (**B**)

After quantifying the amount of Ag NPs in the initial bio-accessible fraction and after 2 h of perfusion in the OoC, it was found that cellular transport of Ag NP standards depends on their size distribution. Consequently, low cellular transport (18 ± 7%) was observed for the 60 nm Ag NP standard (Fig. 2A) contrasting with the value obtained for the 40 nm Ag NP standard (cellular transport of 77 ± 8%, Fig. 2A).

Regarding the size distribution of Ag NPs (see Table 4), it was found that the mean size remained unchanged for the 40 nm standards (bio-accessible fraction before and after conditioning for cell viability). However, the mean size decreased for the 60 nm Ag NPs when the bio-accessible fraction was treated for cell culture and after cellular transport. This result is consistent with the low cell uptake of 60 nm Ag NPs, indicating higher dissolution of this material during transport.

**Table 4:** Mean sizes of Ag NPs and TiO_2_ NPs in standards and samples before and after the in vitro bio-accessible and cellular transport within the OoC

	Ag NPs mean size (nm)^a^			
Standard/sample	Standard/Sample extract	Bio-accessible fraction^c^	Bio-accessible fraction^d^	Bioavailable fraction
60 nm Ag NPs	59 ± 5^b^	53 ± 4	43 ± 1	42 ± 1
40 nm Ag NPs	41 ± 3^b^	35 ± 3	35 ± 1	37 ± 1
Small size sugar pearls	77 ± 2	30 ± 4	33 ± 2	34 ± 2
Medium size sugar pearls	60 ± 2	34 ± 2	30 ± 3	33 ± 2
Dulse (*Palmaria palmata*)^d^	22 ± 1	25 ± 1	41 ± 1	42 ± 1
Sea lettuce (*Ulva sp.*)^e^	31 ± 5	30 ± 1	48 ± 2	49 ± 1
Mussel (*Mytilus edulis*)^e^	39 ± 1	38 ± 3	52 ± 1	55 ± 1
	TiO_2_ NPs mean size (nm)^a^			
100 nm TiO_2_ NPs	100^b^	286 ± 11	285 ± 17	278 ±15
50 nm TiO_2_ NPs	50^b^	148 ± 4	159 ± 7	173 ± 4
E171 (nano-/micro-titanium)	–-^f^	138 ± 12	64 ± 11	73 ± 7
Dulse (*Palmaria palmata*)^e^	86 ± 5	77 ± 10	78 ± 1	80 ± 7
Sea lettuce (*Ulva sp.*)^e^	88 ± 2	81 ± 2	79 ± 2	73 ± 6
Mussel (*Mytilus edulis*)^e^	96 ± 2	72 ± 2	67 ± 5	75 ± 7

^a^ *n* = 6; ^**b**^ given by the manufacturer; ^c^ bio-accessible fraction after the in vitro bio-accessibility process; ^d^ bio-accessible fraction (in vitro bio-accessibility process) after conditioning for cell culture; ^e^ seafood exposed to Ag NPs/TiO_2_ NPs under controlled laboratory conditions; ^f^ not calculated

Conversely, cellular transport of TiO₂ NPs of different sizes was found to differ significantly from previous observations with Ag NPs (Fig. 2B), being observed a higher uptake (51 ± 3%) for large TiO₂ NPs (mean size 100 nm) than for 50 nm ones (8 ± 1%). These results are consistent with the cellular transport observed for the food additive E171 (micro/nano titanium dioxide), which has a broad size distribution (138 ± 12 nm) and similar cellular transport (47 ± 9%) to that observed for 100 nm TiO₂ NPs.

**Fig. 3:**
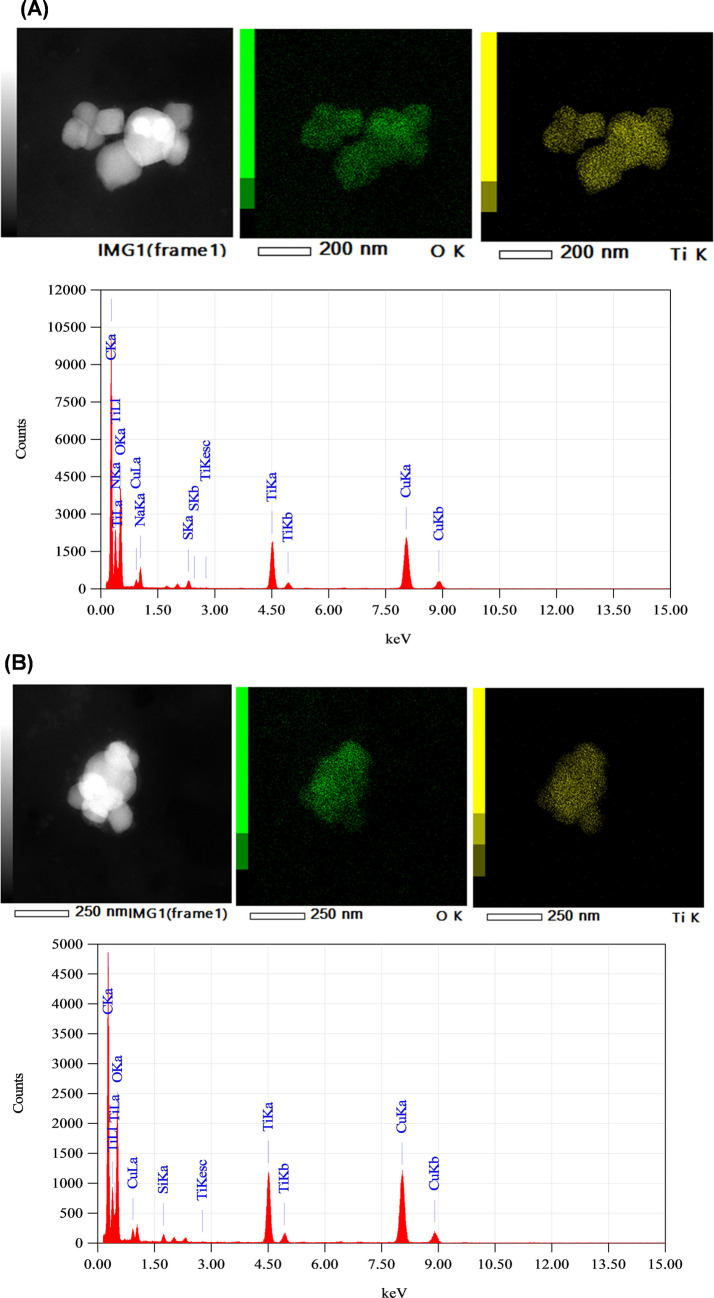
HRTEM images and EDX spectra for conditioned bio-accessible fractions from food additive E171 before (**A**) and after (**B**) perfusion

Table 4 also shows that the mean size of the 100 nm TiO₂ NPs in the bio-accessible digest remains constant after conditioning and cellular transport. However, in the case of the 50 nm TiO₂ NPs, the mean size after cellular transport was slightly higher than the mean sizes in the bio-accessible fractions. In the case of food additive E171, the mean size of TiO₂ NPs after conditioning of the bio-accessible fraction and cellular transport was found to be almost half the original size (from initially 138 ± 12 nm to 64 ± 11 and 73 ± 7 nm before and after perfusion, Table 4). Small sizes of the conditioned bio-accessible fractions of food additive E171 (before and after perfusion) can also be observed by HRTEM (Fig. 3). These images show a high degree of agglomeration of TiO₂ NPs.

#### Cellular transport of Ag NPs in confectionary products containing the food additive E174

Figure 2A shows that cellular transport is higher for confectionery products containing the food additive E174 in sample 1 (small sugar beads, 44 ± 9%) than in sample 2 (medium sugar beads, 21 ± 6%). Different bio-accessibility ratios were also found for the two samples (see Sect. "Bio-accessibility of Ag NPs from confectionary products containing the food additive E174"), which are likely to be related to the composition of the sugar pearls, given that they come from different manufacturers. In terms of size, the mean size of both confectionery products remained unchanged in the original and treated bio-accessible digest, as well as in the digest after perfusion (close to 30 nm in all cases; see Table 4). Using HRTEM (Figure S3, ESM), a high degree of agglomeration of small (less than 50 nm) Ag NPs was observed. In this regard, it is known that the aggregation of silver nanoparticles is induced by high salt concentrations [42].

#### ***Cellular transport of Ag NPs and TiO***_***2***_*** NPs in seafood***

Despite the different nature of the two analysed cocked seaweed samples (red and green), the cellular transport of Ag NPs (Fig. 2A) was found to be similar (55 ± 4% and 48 ± 5% for dulse and sea lettuce, respectively). Additionally, the in vitro assay for the mussel sample revealed a similar transport percentage (54 ± 9%) to that obtained for the seaweeds.

**Fig. 4:**
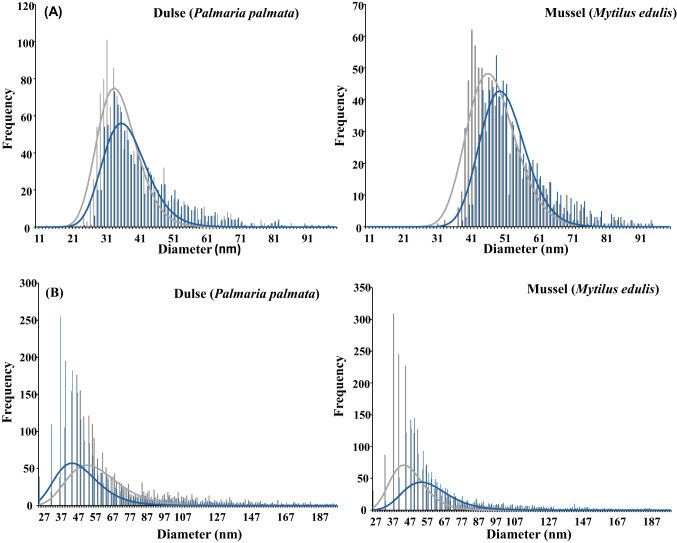
Particle size distribution for Ag NPs (**A**) and TiO_2_ NPs (**B**) in cocked dulse and mussel before (grey line) and after (blue line) OoC perfusion

The treatment of the bio-accessible fractions (mainly osmolarity adjustment by adding concentrated NaCl solutions) for cell viability in the OoCs could explain the difference in size observed in the treated bio-accessible fractions (the mean size increased by approximately 10 nm compared to the untreated bio-accessible fractions; see Table 4). Osmolarity adjustment of seafood bio-accessible fractions required a higher volume of 5.0 M NaCl than used for Ag NP standards and confectionery, which leads to a high degree of agglomeration of Ag NPs (HRTEM-EDX images in Figure S2 and S3, ESM). However, the mean sizes (see Table 3) and size distributions (see Fig. 4A for dulse and mussel) of the treated bio-accessible fraction before and after perfusion in the OoC devices were found to be quite similar. These results are in good agreement with those observed for Ag NPs standards.

In general, the results of the cellular uptake of Ag NPs in seaweed and molluscs differ when the conditions are dynamic rather than static. For example, some studies on seaweed indicate very low cellular uptake of Ag NPs in cooked seaweed under static conditions (less than 1%) [22, 36], contrasting with results obtained under dynamic conditions (over 50%). However, in the case of Ag NPs in cooked shellfish, cellular uptake under static conditions was found to be in the range of 2–22% for various species of shellfish [23], and between 57 and 65% for Japanese carpet shell [24]. These values are consistent with those obtained under dynamic conditions for similar foods.

**Fig. 5:**
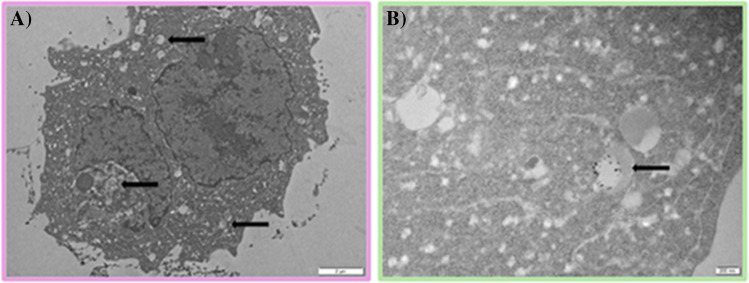
TEM image of a Caco-2 monolayer exposed to a mussel digest containing TiO₂ NPs: (**A**) Scale: 2 µm; (**B**) Scale: 200 nm

Similar percentages were obtained for the cellular transport of TiO₂ NPs in cocked seaweed (35 ± 9% and 35 ± 5% for dulse and sea lettuce, respectively), whereas cellular transport was negligible in mussels (Fig. 2B). This insignificant cellular uptake in mussels cannot be attributed to the size of the TiO₂ NPs, since the mean sizes in the bio-accessible mussel digest were much lower than those measured in the digests of TiO₂ NP standards and the food additive E171 (Table 4). Non-cellular transport could be related to the digested mussel matrix, which could favour nanoparticle retention inside the cells. To confirm this, TEM images of cellular cross-sections were obtained. Figure 5A shows a circular structure measuring approximately 15 µm in diameter, which is consistent with the size of Caco-2 cells (ranging from 10 to 40 µm in diameter) as observed using bright-field microscopy of cell cultures. Within the cytoplasm, spherical structures with diameters between 200 and 400 nm can be seen. This is consistent with the formation of intracellular vesicles, which typically range in size from 50 to 1000 nm. Higher magnification of these structures (Fig. 5B) reveals smaller black dots measuring 20–40 nm, as well as aggregates of these dots. These dots and aggregates are consistent in size with the TiO₂ NPs to which the cells were exposed [43].

Based on these results and ICP-MS data, it can be confirmed that TiO₂ NPs can interact with and penetrate the cell monolayer. This is in line with previous reports on the internalisation of NPs in Caco-2 cells [23, 44, 45].

Regarding the size distributions (see Table 4 and Fig. 4B for dulse and mussel), the mean sizes of the bio-accessible fraction treated before and after perfusion in the OoC devices were found to be quite similar. These results are in good agreement with those observed for TiO₂ NPs standards.

The cellular uptake rates of TiO_2_ NPs in seaweed under dynamic conditions (approximately 35%) are higher than the values reported in the literature for seaweed and other marine foods, which range from 1.6 to 2.7% [36]. Regarding mussels, data from the literature show cellular uptake of TiO_2_ NPs of less than 1.4% [36], consistent with the value obtained under dynamic conditions. In contrast, other studies on seafood report high cellular uptake values: 76% in cooked Japanese carpet shell and 100% in sea bream [24] and variable percentages (17–82%) in a variety of raw shellfish [23].

## Conclusions

In this study, we optimised the operating conditions of a μ-Slide I Luer 3D (from Ibidi) commercial OoC device to mimic human intestinal absorption of nanomaterials in foodstuff. The dynamic approach used reflects actual physiological conditions in the intestine more closely and could provide a more realistic estimation of the bioavailability of pollutants (in our case, Ag NPs and TiO_2_ NPs) than conventional static approaches. In the case of bioavailability, a parameter established in our study through cellular transport, these conditions have produced values higher than those reported for static bioavailability as-says for Ag NPs and TiO_2_ NPs in seaweed. Conversely, cellular transport values for both types of nanoparticles in molluscs were similar to those reported under static conditions, indicating the significant impact of the food matrix on the process. Although our study focused on two types of nanoparticles, the procedure can be used to assess the cellular transport of essential and toxic metals, as well as other nutrients and organic contaminants in food. This makes it a powerful and realistic tool for evaluating bioavailability.

However, the lack of standardised procedures must be emphasised, as this makes it difficult to establish comparisons with results reported in the literature. This is particularly important since, in addition to the published results based on static model approaches, a variety of OoC devices are currently on the market. Therefore, studies must be expanded to include these devices, which reflect cellular transport under dynamic conditions, in order to reach a consensus on the operational conditions required for bioavailability studies.

## Supplementary Information

Below is the link to the electronic supplementary material.

Supplementary file1 (DOC 2463 KB)

## Data Availability

No datasets were generated or analysed during the current study.
