# Supplementary material for: Use of organ-on-chip devices and single particle ICP-MS for assessing dynamic in vitro bioavailability of silver and titanium dioxide nanoparticles from foodstuff
Source: Mikrochim Acta. 2026 Apr 24;193(5):342. doi: 10.1007/s00604-026-08062-y (PMC13109290; doi:10.1007/s00604-026-08062-y)
Supplement: Supplementary file 1 — Supplementary file1 (DOC 2463 KB) [file 604_2026_8062_MOESM1_ESM.doc]

Electronic Supplementary Information

**Use of organ-on-chip devices and single particle ICP-MS for assessing dynamic *in vitro* bioavailability of silver and titanium dioxide nanoparticles from foodstuff**

Osvaldo Beltrán-Osuna, Juan José López-Mayán, Lucía Gómez-Cibeira, Oier Jurado-Martín, Alejandro Ogando-Cortés, Raquel Domínguez-González, Pablo Taboada-Antelo, Pilar Bermejo-Barrera, Antonio Moreda-Piñeiro

**1. Nanoparticles determination by spICP-MS**

The determination of NPs (i.e. particle number concentration and size distribution) was performed using spICP-MS (NexION 2000 with Syngistix™ Nano Application software, PerkinElmer, Massachusetts, USA) under the instrumental conditions given in Table 1. A commercial multielement solution (Set up NexION Solution, 10 µg L^-1^ of Be, Ce, Fe, In, Li, Mg, Pb, and U, PerkinElmer) was used to optimise the instrumental conditions just before the sample flow rate was assessed. Ultrapure water (18 MΩ cm resistivity, obtained from a Milli-Q® IQ7003, Millipore Co., Bedford, MA, USA) was pumped at the established peristaltic pump rate for 5.0 minutes, after which the weight loss was measured. The sample flow rate was consistently close to 0.10 mL min^-1^ (0.107±0.093 mL min^-1^). The transport efficiency (TE%) was assessed daily using an Au NP suspension (51 nm, 1.1×10^5^ NPs mL^-1^) prepared from PEG-carboxyl Au NPs in aqueous 1.0 mM citrate (51.0±1.9 nm by TEM, 15.3 ng mL^-1^, 1.10×10^7^ NPs mL^-1^, data given by the manufacturer) from nano Composix (San Diego, CA, USA) and an aqueous ionic Au calibration (0.5-3.0 µg L^-1^) prepared from AuCl_4_ stock standard solutions (1000 μg mL^-1^) from Merck (Darmstadt, Germany). The Syngistix™ Nano Application automatically calculates the TE, which varied between 8 and 12%. Calibrations were then performed with ionic Ag and Ti solutions within the 1-5 µg L^-1^ range prepared from 1000 μg mL^-1^ in 1% de HNO_3_ v/v Ag NO_3_ (Merck) and (NH_4_)_2_TiF_6_ (PerkinElmer) stock standard solutions. Kinetic energy discrimination (KED) mode was used to assess the Ag NPs (with He as the collision gas, as shown in Table 1), whereas dynamic reaction cell (DRC) mode (with NH_3_ as the collision gas and an ammonia cluster ^48^Ti(NH)(NH_3_)_4_ at m/z 131 [[[1]](#endnote-2)], see Table 1) was used for TiO_2_ NPs determinations.

**Table 1**. Operational parameters for spICP-MS measurements

| Parameter/Component | Ag NPs | TiO_2_ NPs |
| --- | --- | --- |
| Nebulizer type | Concentric Meinhard™ | Concentric Meinhard™ |
| RF power (W) | 1600 | 1600 |
| Ar Plasma gas flow rate (L min^−1^) | 15 | 15 |
| Ar auxiliary gas flow rate (L min^−1^) | 1.2 | 1.2 |
| Ar nebulizer gas flow (L min^−1^) | 1.15 | 1.15 |
| Ammonia flow rate (mL min^−1^) | --- | 1.0 |
| Helium flow rate (mL min^−1^) | 4.5 | --- |
| Analyte (m/z) | Ag (107) | Ti (131) |
| Ion-product registered | --- | ^48^Ti(NH)(NH_3_)_4_ |
| Mass fraction (%) | 100 | 59.9 |
| Density (g cm^-3^) | 10.49 | 4.23 |
| Dwell time (μs) | 50 | 100 |
| Reading time (s) | 100 | 100 |
| Acquisition time (s) | 100 | 60 |
| Mode | KED | DRC |
| Replicates | 3 | 3 |

The limit of detection (LOD) for determining NPs by spICP-MS was calculated using the 5σ criterion proposed by Laborda et al. [36] obtaining an LOD for number concentration of 1.3×10^6^ and 1.4×10^7^ NP L^-1^ for Ag NPs and TiO_2_ NPs respectively. Regarding the LOD in size, the Syngistix™ Nano software provided LODs of 12 and 22 nm for Ag NPs and TiO_2_ NPs respectively.

**2. Sample pretreatment procedure for NPs extraction**

**2.1 Ag NPs and TiO_2_ NPs isolation from mussel**

Enzymatic hydrolysis based on pancreatin and lipase exposure was used to extract Ag NPs and TiO₂ NPs from 1.0 g of wet homogenised tissue. The extraction of both type of NPs was performed using 3 g L⁻¹ and 8 g L⁻¹ of pancreatin and lipase (porcine pancreatin and lipase from *Candida rugose,* Sigma Aldrich, Osterode, Germany), respectively, in a 0.2 M NaH₂PO₄/0.2 M NaOH buffer solution at pH 7.4 (prepared from ACS reagents from Sigma Aldrich). Extraction was performed overnight at 37 °C and 150 rpm in a Boxcult temperature-controlled chamber equipped with an orbital-rocking shaking platform (Stuart Scientific, Surrey, United Kingdom). Each sample was subjected to the enzymatic digestion in triplicate, and reagent blanks were also prepared and analysed in duplicate. The obtained extracts were filtered using Minisart NML hydrophilic non-sterile 5.0 µm cellulose syringe filters (Sartorius, Goettingen, Germany) before spICP-MS analysis.

**2.2 Ag NPs isolation from seaweed**

The procedure for extracting Ag NPs from seaweed involved mixing 0.5 g of homogenised wet seaweed tissue with 7.0 mL of a solution containing 2.0 mM citric acid and 2.0 mM trisodium citrate (pH 4.5) prepared from citric acid (Panreac, Barcelona, Spain) and trisodium citrate di-hydrated (Merck), followed by ultrasonication at 20% amplitude for 2.5 min (with intermittent 1.0-second pulses, for a total pre-treatment time of 5.0 min) using a VibraCell™ VCX 130 V ultrasound probe (Sonics Newtown, CT, USA). After this step, 2.0 mL of a solution containing Mazerozyme® R-10 (Merck, Darmstadt, Germany) at 25 g L^-1^ was added and enzymatic hydrolysis proceeded under controlled temperature (37 °C) and constant orbital rocking at 150 rpm for 6 h. The samples were treated in triplicate and several reagent blanks were prepared. Determinations were performed by spICP-MS after filtration of the extracts using 5.0 µm cellulose syringe filters.

**2.3 TiO_2_ NPs isolation from seaweed**

TiO_2_ NPs were extracted from seaweed by using an ultrasound assisted alkaline extraction procedure (Raypa UCI-150 water bath ultrasonic cleaner, R. Espinar S.L., Barcelona, ​​Spain) in accordance with the method described by López-Mayán et al. [[[2]](#endnote-3)]: 1.0 g of seaweed was mixed with 7.5 mL of 2.5% (v/v) tetramethylammonium hydroxide (TMAH), prepared from TMAH 25 %(v/v) in water (Merck) under ultrasound (45 kHz, 80 W) for 2 h. All samples were treated in triplicate, with at least two reagent blanks prepared. All extracts were finally filtered using 5.0 µm cellulose syringe filters to remove solids before spICP-MS analysis.

**2.4 Ag NPs isolation from confectionery products containing the food additive E174**

The enzymatic hydrolysis procedure using Mazerozyme® R-10, which was previously described for the isolation of similar Ag NPs from seaweed [[[3]](#endnote-4)], was applied to pre-treat confectionery products. In this case, the sugar pearls were lightly crushed using a glass mortar and pestle, and the sample mass fixed at 0.50 g. Each sample was treated in triplicate and at least two reagent blanks were prepared for each case. Finally, a filtration stage using 5.0 µm cellulose syringe filters was carried out before measurements were taken.

**3. Culinary treatment**

The culinary treatment consisted of boiling 10 g of mussel and seaweed in 600 mL of pre-heated ultrapure water (controlled with a thermometer within the 90–100 °C range) for 10 min. The cooking water was then removed by gravity filtration, after which the cooked samples were placed on Petri dishes, and left to cool at room temperature before being dried in an oven at 30-40°C. The calculated moisture percentage was 61% for mussels and 78 and 75% for *Palmaria palmata* and *Ulva sp*., respectively. The dried samples were stored in polyethylene tubes at -18°C prior to use.

**4. *In vitro* gastrointestinal digestion**

To perform the *in vitro* digestion 0.5 g solid sample (sugar pearls, seaweed, or mussel) were dispersed in 10 mL of ultrapure water were placed in Erlenmeyer flasks. In the case of nanoparticle suspensions 10 mL of the NP suspensions were treated. The Ag NPs standards were from nano Composix and consisted of sodium citrate-stabilized Ag NPs of 41±3 nm by TEM, 0.021 mg mL^-1^, 5.7×10^10^ NPs mL^-1^, in aqueous 2.0 mM citrate; and sodium citrate-stabilized Ag NPs of 59±5 nm by TEM, 0.021 mg mL^-1^, 1.9×10^10^ NPs mL^-1^ (characterization given by the manufacturer). Dispersions of 50 and 100 nm TiO_2_ NPs (15 % (w/w) in ethanol) were from US Research Nanomaterials (Houston, TX, USA). Food-grade additive E171 (titanium dioxide) was from Minerals-Water (Rainham, United Kingdom).

The pH of all standard/food dispersions was then adjusted to 2.0 (gastric pH) using 0.1M HCl (prepared from 35% (v/v) hydrochloric acid from Merck) after a 10 min stabilization period (pH50+DHS pH-meter, Instruments XS, Carpi Mo, Italy). Next, 0.0725 g of simulated gastric solution prepared in 0.1 M HCl and containing 160 g L^-1^ of pepsin (porcine pepsin from Sigma Aldrich) was added. The flasks were then covered with Parafilm® and placed in an incubation chamber at 37 °C with shaking at 150 rpm for 2 h (gastric digestion). Once this stage was complete, the enzymatic activity was stopped by placing the flask in an ice bath. At this point, the gastric digest was stopped, and the pH was adjusted to 7.0 (intestinal pH) with 0.1 M NaOH. After adjusting the pH, 5.0 mL of simulated intestinal solution prepared in 0.1 M of NaCO_3_ and containing 4 g L^-1^ of pancreatin and 2.58 g L^-1^ of sodium taurocholate (96% hydrated sodium taurocholate, Alfa Aesar, Kandel, Germany) was added. The flasks were then incubated at 37 °C with shaking at 150 rpm for 2 h (intestinal digestion). Once intestinal digestion was complete, the enzymatic activity was stopped in an ice bath, after which the digest was centrifuged at 3.900 rpm and at 4 °C for 30 min (Sigma 2K15 centrifuge with rotor 12141, (Sigma GmbH, Osterode, Germany). The resulting supernatant (bio-accessible fraction) was collected and filtered using 5.0 µm cellulose syringe filters. The *in vitro* digestion process was performed in triplicate for each sample, and a reagent blank was also prepared in triplicate for each session.

**5. *In vitro* gastrointestinal digestion**

**5.1 Bio-accessible extract conditioning for cell-based assays**

To perform cellular assays, the samples (bio-accessible fractions) must be conditioned to guarantee cell viability. Therefore, 5.0 mL of bio-accessible fractions (see section 2.5) were heated at 90 °C for 15 min to inactivate the enzymes. After enzyme inactivation, 50 µL of a 100 mg mL^-1^ glucose solution (prepared from D (+)-glucose, Merck) was added, and the osmolarity was adjusted to 280–300 mOsm kg^-1^ with a 5.0 M NaCl solution (prepared from sodium chloride, Merck) using a Semi-Micro Osmometer K-7400S (KNAUER, Berlin, Germany).

**5.2 Cell culture**

The Caco-2 cells (colon cancer cells Caco-2 were purchased from Cell Biolabs, San Diego, CA, USA) were cultured in Dulbecco's Modified Eagle Medium (DMEM), which was supplemented with 10% (v/v) fetal bovine serum (FBS), 1% (v/v) penicillin/streptomycin and 1% (v/v) essential amino acids (Thermo Fisher Scientific, Waltham, MA, USA). The cells were then incubated at 37 °C in an atmosphere containing 5.0% CO₂ and 90% humidity (icoMed Memmert controlled atmosphere incubator, Schwabach, Germany).

**5.3 Microfluidic chips**

The μ-Slide I Luer 3D microfluidic chip (Ibidi, Gräfelfing, Germany) consisted of a single channel and three wells in which cells could be cultivated. Table 2 lists data regarding the outer dimensions of the OoC, the dimensions of the wells and the channel parameters. Similarly, Figure 1 shows a diagram of the device and how it is connected to the perfusion system.

**Table 2**. µ-Slide I Luer 3D specifications (from https://ibidi.com/channel-slides/250--slide-i-luer-3d.html).

| Outer dimensions (w × l) | 25.5 × 75.5 mm |
| --- | --- |
| Number of wells | 3 |
| Volume of wells | 16 µl |
| Well dimensions (w × l) | 5.4 × 4.0 mm |
| Well height (without channel) | 0.8 mm |
| Growth area per well | 0.21 cm² |
| Coating area per well | 0.34 cm² |
| Channel width | 5.0 mm |
| Channel volume (without wells) | 150 µL |
| Channel height (without well) | 0.6 mm |
| Adapters | Female Luer |
| Volume per reservoir | 60 µL |

**Figure 1**. Schematic representation of the µ-Slide I Luer 3D chip and experimental setup with the perfusion unit

First, each well was filled with 16 µL of a collagen solution (2.0 mg mL^-1^ according to the supplier's specifications) prepared from collagen type I from rat tail (Ibidi) and allowed to polymerise at 37 °C for 30 min under 5.0% CO₂ and 90% humidity. After collagen polymerisation, 300 µL of a cell suspension at a concentration of 1.66 × 10⁶ cells mL^-1^ was added, and the cells were allowed to adhere to the collagen layer surface for 5 h before being exposed to flow conditions. Once the cells had adhered, the chip was connected to the microfluidic system's perfusion set (Ibidi), which was filled with DMEM culture medium supplemented with 10% (v/v) foetal bovine serum, 1.0% (v/v) penicillin/streptomycin, and 1.0% (v/v) essential amino acids, at a pressure of 5.0 mbar to achieve a shear stress of 0.2 dyne cm^-^². The cells were then allowed to grow in this culture environment at 37 °C with 5% CO₂ and 90% relative humidity until they reached 100% confluence on the chip surface and formed a cellular monolayer.

**5.4 Optimisation of OoC operating conditions**

The optimisation of operating conditions for microfluidic chips includes the evaluation of shear stress, the time required to obtain a cellular monolayer and cell viability under flow conditions.

Evaluation of shear stress

The wall shear stress in a μ-Slide microchip depends on the flow rate and the viscosity of the perfusion medium. As the software used does not contain the preset parameters for this type of chip, the appropriate flow rates the μ-Slide I Luer 3D device was calculated in accordance with the manufacturer’s recommendations (Equation 3) [[[4]](#endnote-5)]


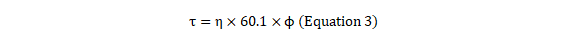


where τ is the wall shear stress, η is the viscosity of the perfusion medium, and Φ is the applied flow rate.

The range of shear stress along the intestine has been established in the range of 0.02–0.20 dyn cm⁻², and a value of 0.02 dyn cm⁻² was adopted for this calculation. In addition, the viscosity of the perfusion medium was 0.0072 dyn s⁻¹ cm⁻¹, resulting in a required flow value of 0.046 mL min⁻¹. To determine the necessary pressure to achieve this shear stress, a linear correlation was established by measuring the flow rate (Φ) in mL min^-1^ at different pressures (P) in mbar within the perfusion system. The slope of the resulting curve (Φ = 0.009P + 0.0247, R² = 0.9891) was used to calculate the pressure required to achieve a flow rate of 0.046 mL min^-1^, which was found to be 5.15 mbar.

Time required to obtain a cellular monolayer

The time required to obtain a cell monolayer on the surface of the microfluidic chips was optimised under the previous perfusion conditions. Cell growth (initial cell population of 1,500,000) was visually monitored at different time points (0, 12, 24, 48 and 72 h) using optical microscopy. As shown in Figure 2A, after 5 h of seeding in static conditions, cells, approximately 30 µm in size, can be seen grouped in a manner reminiscent of Caco-2 cells. Figure 2B shows the cells after 5 h of seeding in flow conditions, confirming that the cells can withstand these conditions and adhere to the chip surface. After 12 h of monitoring under flow (Figure 2C), it is evident that the cells have predominantly covered the surface, leaving only a few free spaces. This indicates that the growth process was allowed to continue for an extended period. After 24 h (Figure 2D), the cell monolayer displays a more compact morphology due to the reduction in the spaces between cells. However, some free spaces are still evident. After 48 h (Figure 2E), the cells had covered approximately 90% of the surface, and after 72 h (Figure 2F), the cells had completely covered the surface, indicating the full formation of the monolayer. These results suggest that 72 h is the optimal incubation time for achieving complete monolayer formation, and this timeframe was therefore selected.


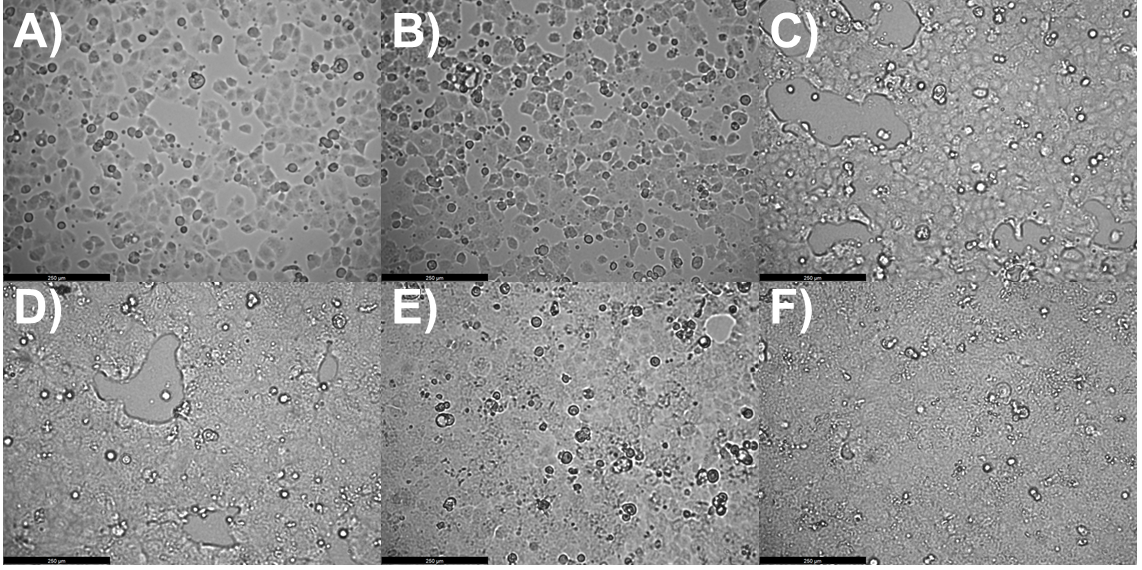


**Figure 2**. Optical microscopy images (scale 250 µm) of colon cancer Caco-2 cells seeded on the Ibidi µ-Slide I Luer 3D microfluidic chip: Cells 5 h after being seeded without flow (A), and cells 5 (B), 12 (C), 24 (D), 49 (E), and 72 h (F) after being seeded under flow conditions

Cell viability under flow conditions

Cell viability under flow conditions was assessed using a membrane integrity assay. Figure 3A shows an optical image in which a homogeneous and compact cell monolayer, consistent with previous results and confirming that 72 h is sufficient time to form the monolayer. Similarly, Figure 3B shows the same field of view after light irradiation at a wavelength of 517 nm to detect green fluorescence from previously stained cells with Calcein AM. Almost all of the cells exhibit fluorescence, indicating that their cell membranes remain intact so that they are alive. Conversely, when cells are irradiated at a wavelength of 617 nm (Figure 3C), hardly any fluorescence from ethidium homodimer-1 is observed, indicating that the number of cells with compromised cell membranes is almost null.


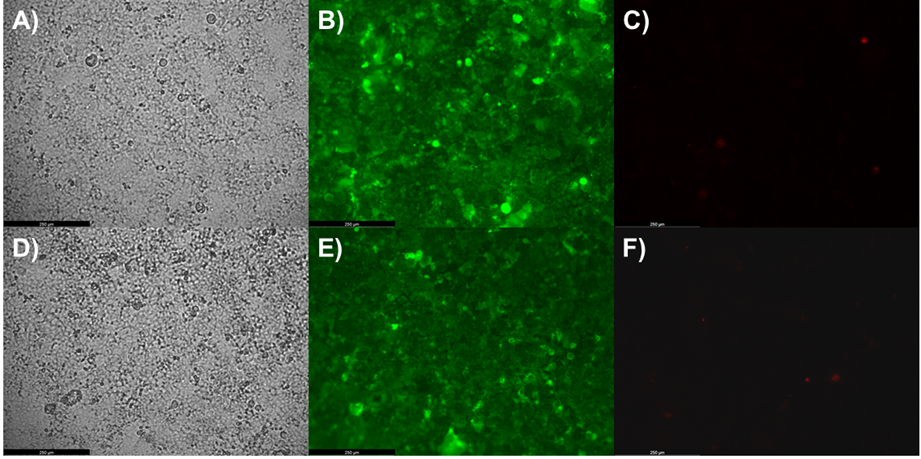


**Figure 3**. Optical microscopy images of cell monolayer after 72 h under flow conditions (A) and after applying the live/dead^TM^ staining using calcein AM and irradiation at 517 nm (B) and propidium iodide and irradiation at 617 nm (C). Optical microscopy images of cell monolayer after 96 h under flow conditions (D) and after applying the live/dead^TM^ staining using calcein AM and irradiation at 517 nm (E) and propidium iodide and irradiation at 617 nm (F). Scale 250 µm.

Since 72 h are required for cell monolayer formation, it is also necessary to verify the integrity of the cell monolayer after this time. Figure 3D–F shows microscopy images taken after 96 h. The results demonstrate an intact, compact cell monolayer (Figure 3D) with full cell viability (Figures 3E,F) after 96 h under flow conditions. Therefore, exposure assays (the addition of the bio-accessible fraction to assess bioavailability) can be performed without concerns about cell viability within the time frame of the experiments.

**5.5 Cell membrane integrity**

A Live/Dead® Viability Assay (Invitrogen, Eugene, OR, USA) was performed to determine the extent of cell membrane integrity in cells under flow conditions. This assay uses a mixture of the fluorescent dyes calcein AM and ethidium homodimer-1. The former is converted into the fluorescent compound calcein by intracellular esterases in viable cells (cells are now capable of emitting bright green fluorescence). Ethidium homodimer-1 enters cells with compromised membranes (i.e. damaged cells) and produces bright red fluorescence by intercalating into DNA.

After 72 and 96 h of incubation, the stained cells were observed under a fluorescent microscope (Leica DMI6000 B, Wetzlar, Germany) with the appropriate fluorescent filter cubes at 10X magnification. Images obtained using different fluorescent filters were merged using ImageJ software (NIH, Bethesda, MD, USA).

**6. HRTEM-EDX analysis of bio-accessible/transport fractions**

The bio-accessible fractions, both before and after perfusion, underwent a cleaning/pre-concentration stage prior to HRTEM-EDX analysis. Firstly, the fractions (with variable volumes between 1.2 and 3.0 mL) were dialyzed using Slide-A-Lyzer dialysis cassettes (3.5 kDa MWCO) from Thermo Scientific (Rockford, IL, USA) and ultrapure water (300 mL) as the accepting solution. A first dialysis step was performed for 2 h, followed by a second step involving fresh ultrapure water overnight. The dialysed fractions were then centrifugally filtered (30 kDa MWCO Amicon Ultra-4 centrifugal filter units, Millipore, Cork, Irland) using a fixed-angle rotor optimised for g-force (2364×g) in a centrifuge Sigma 2K15 centrifuge (Sigma GmbH), for 15 min, resulting in approximate final volumes within the 125–200 µL range.

The cleaned concentrate was then recovered, 10 µL of which were dropped onto a copper grid and left to air-dry at room temperature prior to HRTEM-EDX analysis in a JEM-F200CF-HR high resolution transmission electron microscope coupled with a JEOL JED-2300T energy-dispersive X-ray spectroscopy (JEOL, Tokyo, Japan).

**7. Preparation of Caco-2 cell sections and TEM analysis**

After 2 h of exposure to the Caco-2 cell monolayer, the Ag NP digest was removed from the microfluidic chip channel and the cells were trypsinised. Initially, 200 µL of PBS (Millipore) was added to the microfluidic chip channel three times to rinse the cell monolayer. Then, trypsin (obtained from trypsin from porcine pancreas, Sigma-Aldrich) was added to the channel and the chip was placed in an incubator at 37 °C for 5 min. The added trypsin volume was then collected and 200 μL of fresh DMEM was added to ensure all the cells were recovered and to neutralise the effect of the trypsin. The cell suspension was then centrifuged at 1500 rpm for 4 min. The resulting cell pellet was fixed with 500 μL of 1.5% (w/v) glutaraldehyde (Grade II, 25 % in ultrapure water, Sigma-Aldrich), embedded in an agar pellet and fixed again with 1% (w/v) osmium tetroxide in 0.1 M cacodylate buffer (reagents from Sigma-Aldrich). Finally, a pellet was formed by adding eponate (Ted Pella, Redding, CA, USA) and cell sections were obtained using an ultramicrotome (UltraCut S ultramicrotome, Leica Microsystems GmbH, Wetzlar, Germany). The cell sections were further analysed TEM (JEOL JEM-1011).

**Figure S1**. Bio-accessibility of Ag NPs in Ag NPs standards (60 and 40 nm), confectionery products (sugar pearls) and exposed seaweeds (dulse and sea lettuce) and mussels (A), and bio-accessibility TiO_2_ NPs in TiO_2_ NPs standards (100 and 50 nm), food additive E171, and exposed seaweeds (dulse and sea lettuce) and mussels (B)

**Figure S2**. HRTEM images and EDX spectra for conditioned bio-accessible fraction from small size sugar pearls containing food additive E174 before (A) and after (B) perfusion

**Figure S3**. HRTEM images and EDX spectra for conditioned bio-accessible fraction from mussel before (A) and after (B) perfusion

**References**

1. [?] Suárez-Oubiña C, Herbello-Hermelo P, Bermejo-Barrera P, et al (2022) Single-particle inductively coupled plasma mass spectrometry using ammonia reaction gas as a reliable and free-interference determination of metallic nanoparticles. Talanta 242:123286. https://doi.org/10.1016/j.talanta.2022.123286. [↑](#endnote-ref-2)
2. [?] López-Mayán JJ, del-Ángel-Monroy S, Peña-Vázquez E, et al 2022) Titanium dioxide nanoparticles assessment in seaweeds by single particle inductively coupled plasma–Mass spectrometry. Talanta 236:122856. https://doi.org/10.1016/j.talanta.2021.122856. [↑](#endnote-ref-3)
3. [?] López-Mayán JJ, Álvarez-Fernández B, Peña-Vázquez E, et al. (2022) Ultrasonication followed by enzymatic hydrolysis as a sample pre-treatment for the determination of Ag nanoparticles in edible seaweed by SP-ICP-MS. Talanta 247:123556. https://doi.org/10.1016/j.talanta.2022.123556 [↑](#endnote-ref-4)
4. [?] Ibidi Application Note 11. Shear stress and shear rates for ibidi μ-slides based on numerical calculations (2022) ibidi GmbH, Version 6.1. <https://ibidi.com/img/cms/downloads/an/AN11_Shear_stress.pdf> (accessed September 2025). [↑](#endnote-ref-5)
